# Having to manage: culturally and linguistically diverse mothers’ lived experiences with sustained nurse home visiting programs

**DOI:** 10.1186/s12913-023-09315-9

**Published:** 2023-04-11

**Authors:** Mehrnoush Bonakdar Tehrani, Kelly Baird, Suza Trajkovski, Lynn Kemp

**Affiliations:** 1 Translational Research and Social Innovation (TReSI), Ingham Institute for Applied Medical Research, Level 3, 1 Campbell Street, Liverpool, NSW 2170 Australia; 2 School of Nursing and Midwifery, Western Sydney University, Sydney, Australia

**Keywords:** Culturally and linguistically diverse, Child and family health nursing, Limited English proficiency, Migrant, Sustained nurse home visiting

## Abstract

**Background:**

Culturally and linguistically diverse (CALD) mothers with young children with limited English proficiency (LEP) encounter multiple barriers in accessing and engaging with primary healthcare services. The aim of this study was to explore the lived experiences and perceptions of CALD mothers with LEP in receiving child and family health nursing (CFHN) services and sustained nurse home visiting (SNHV) programs.

**Methods:**

Fourteen mothers were interviewed from two large Local Health Districts in Sydney. All interviews were audio-recorded for transcription purposes. Interpretative Phenomenology Analysis (IPA) was used for analysis and the socioecological approach was applied to interpret the data.

**Results:**

CALD mothers with LEP experienced both challenges and facilitators in their access and engagement with CFHN services and SNHV programs that were identified in four themes: managing culture; managing the service system; managing the relationship; and strengths and weaknesses of CFHN services.

**Conclusion:**

The integration of strategies such as building trusting relationships, using female professional interpreters and better understanding of CALD mothers’ cultural practices may address CALD mothers’ needs and facilitate communication. Design and development of model of support involving CALD mothers with LEP in ways that voice their ideas could meet their needs and contribute to better engagement of this vulnerable population with CFHN services and SNHV programs.

**Supplementary Information:**

The online version contains supplementary material available at 10.1186/s12913-023-09315-9.

## Background

Culturally and linguistically diverse (CALD)^1^ mothers with young children with limited English proficiency (LEP) encounter multiple barriers in accessing and engaging with primary healthcare services in comparison with non-migrant or English-speaking migrants in host countries [1, 2]. Previous studies have shown CALD families experienced several challenges including language and communication barriers; cultural incompatibility; social isolation; unfamiliarity with healthcare services; and access issues [3–6]. These barriers contribute to a range of adverse outcomes for CALD mothers and their children, including severe psychological distress during pregnancy, postnatal depression, stillbirth, and prenatal mortality [3, 7].

Limited English proficiency is a significant vulnerability that may create particular issues with CALD families and has an adverse impact in accessing and engaging with health services. In addition, program delivery related issues including access to translated information, low level of health literacy, lack of understanding of the role of CFHNs and trust relationship issues significantly influenced engagement of CALD mothers with services available [5, 6, 8, 9]. In Australia, child and family health nursing (CFHN) services are universal and publicly funded and delivered by specialist child and family health nurses (CFHNs); registered nurses with postgraduate qualifications in child and family health. CFHN services provide a wide range of supports including health and developmental surveillance as well as education and information on parenting support, and prevention services to families with children from birth to five years old. Sustained nurse home visiting (SNHV) programs, in particular, are popular and promising models of service delivery to improve service access and outcomes for families with adversity and/or vulnerable families comprising intensive and sustained visits in the family’s home environment by CFHNs over months or years [10–12].

CFHN services and SNHV programs are relationship-based practices (mother-nurse), and engagement is a core facet in these service systems. Several studies have demonstrated the effectiveness of SNHV for disadvantaged mothers, focusing on positive outcomes related to maternal and child health and development including improved birth health and child development outcomes; improved parent-child relationships; higher immunisation rates; increased breastfeeding duration and reduction in child maltreatment; decreased postpartum depression; reduced child abuse; and reduction in subsequent pregnancies [12–15]. There is some evidence from the Family Nurse Partnership program evaluation in the UK that the fidelity of program delivery differs when operating through interpreters, with participating nurses qualitatively reporting these differences [16]. However, no published research has exclusively examined the lived experiences of CALD mothers with LEP receiving CFHN services and SNHV programs.

Therefore, the needs of CALD mothers with LEP and their children in access to and engagement with CFHN services and SNHV programs could be better realised through examining their lived experiences of service provision.

## Methods

### Aim and guiding questions

This study aimed to explore the experiences of CALD mothers with young children with LEP who are receiving CFHN services and SNHV programs in Australia. The study was directed by following research questions:

1. What are the facilitators to access and engagement with CFHN services, particularly in relation to SNHV?;
2. What are the barriers to access and engagement with CFHN services, particularly in relation to SNHV?;
3. How do mothers perceive the role of nurses delivering CFHN services, particularly SNHV, including any child assessments and referrals during the home visiting period?;
4. What factors do mothers perceive affect the quality of the family-nurse-interpreter relationship?

### Design and research context

An inductive qualitative approach was applied, in which semi-structured interviews were conducted with CALD mothers with young children attending either of two large Local Health Districts (LHDs) in urban Sydney, Australia. Both LHDs are in areas with considerable CALD populations. This study particularly aimed to recruit mothers who speak Arabic, Bengali, Mandarin, Vietnamese, or Nepalese, and were receiving CFHN services and SNHV programs. The five community languages identified are the most commonly spoken within these sites.

### Theoretical framework

The analysis was underpinned by a socioecological model [17]. Applying this theory sensitised the researchers to the individual, interpersonal, institutional, and societal level factors that may influence CALD mothers with young children’ lived experiences of access to and engagement with CFHN services and particularly SNHV programs.

### Recruitment

Prior to data collection process, managers and CFHNs of both clinics were contacted by email and met (via Zoom platform) with the first author to inform them of the proposed study. Subsequently, continual contact was maintained with the CFHNs, and they contacted the first author whenever eligible mothers who were interested in participating in the study attended the clinics. Mothers were invited to participate if they met the following inclusion criteria:

- CALD mothers with LEP (e.g., mothers who require the use of an interpreter or mothers who had some English language capacity, but not proficient English language skills as assessed by the CFHN).
- Aged 18 and over.
- Lived experiences of accessing CFHN services, and SNHV programs within either site.
- Mothers who speak Mandarin, Arabic, Bengali, Nepalese, or Vietnamese.

Homogeneous purposive sampling was used to recruit CALD mothers who met the inclusion criteria outlined for the study. As per the study ethics approval, the CFHNs asked CALD mothers who were receiving services whether they wished to participate in the study and obtained verbal consent to pass their contact details to first author. In addition, at the request of the nurse managers, participants were recruited through direct contact with the first author in CFHN clinic waiting rooms. Subsequently, mothers were informed about the study by the first author (assisted by an interpreter if necessary) who provided further information, including reading through the participant information sheet and consent form. The information sheet and consent forms were published in English. Interpreters were used to translate and explain these to the mothers with limited English proficiency in their own language. For the mothers with some English language capacity, the information sheet and consent forms were read and explained in English. Those mothers stated that they understood the contents of these documents and indicated they did not require interpreters. After obtaining informed consent, the first author arranged a date and time (and location for face-to-face interviews) that was convenient for the mothers to complete the interview. Recruitment continued until thematic saturation had been achieved, defined as the interviews providing sufficient information power to identify a cohesive narrative describing mothers’ experiences in relation to the research questions [18].

### Ethical issues

This study is part of a larger project and ethics approval was obtained from the Sydney Local Health District (SLHD) and South Eastern Sydney Local Health District (SESLHD) Ethics Committees with approval numbers (X17-0376 and HREC/17/RPAH/571; SSA 18/RPAH/116; SSA 18/G/078). Potential participants were provided with an information sheet, written in English, that explained the study’s aims, objectives, and procedures. Participants with LEP were assisted by interpreters to ensure their understanding and willingness to participate in the study. Participants with some English language capacity were offered interpreter assistance if it appeared they did not understand. Participants’ consents were obtained in writing prior to participating in interviews and also verbally at the beginning of interviews by first author. Participants’ confidentiality and anonymity were ensured and maintained by using unique ID number during data analysis and reporting of results.

### Data collection

Semi-structured interviews were conducted face-to-face for the participants with some English language capacity either in a private room at CFHN clinic or in participants homes upon their request, and via teleconferences for the participants with LEP with assistance of female professional interpreters to facilitate communication. One participant preferred her husband to interpret the interview. The interview guide was informed by the socioecological model with questions adapted from previous evaluations by the researchers that explored mothers’ experiences of CFHN services. The interviews took 30–60 minutes and were all audio-recorded. At the completion of each interview the interviewer summarised the interview content to check that the information provided had captured the mothers’ experiences, providing opportunity to add to or change the information provided, and confirmed that the participant was happy for information to be included in the study. After conducting the interviews, all participants were offered a $20 gift coupon to a local market in appreciation for their time. All interviews were conducted, recorded and transcribed by the first author, an experienced qualitative interviewer. Transcripts presented only the English components of the interviews for analysis.

### Analysis

Interpretive Phenomenological Analysis (IPA) [19] was used to understand the unique lived experience of CALD mothers receiving CFHN services and SNHV programs. The analysis started with the first author reading and re-reading the transcripts and listening once more to the recording of the interviews while reading the transcripts in order to familiarise themselves with the data, and inductively identifying codes. The co-authors also read and coded the transcripts and contributed to an emerging catalogue of codes which were subsequently mapped to the socioecological model. The coding process was conducted using NVivo 12 qualitative data management software. Patterns in the codes were observed and emergent themes were developed and connections across themes were made aided by reflection on the socioecological model and its explication of the relationships between factors influencing the participants’ experiences. The themes were labelled in a way to illuminate their essence. Some supportive quotations were selected to illustrate the themes. Superordinant themes were identified that captured patterns of mothers’ experiences. Finally, construction of a cohesive narrative based on the superordinant themes illustrated by participants’ quotes was undertaken to add richness and depth. All authors participated in several meetings to discuss processes of data analysis, and final analysis was based on research team consensus, achieved through team meetings and discussion.

### Reflexivity

The research team have several years experiences of working with children and families in health settings and have conducted previous research with multicultural populations. The first author is an experienced neonatal, paediatric nurse and researcher in public health pursuing a Ph.D, with experiences of working with children and disadvantaged families including refugees. The second author is an experienced qualitative researcher with an experience of working with children and families with adversities. The third author has extensive experience as a neonatal intensive care nurse and researcher and has worked with families during and following neonatal hospital admission and the fourth author is a child and family health nurse and recognised as an international leader in the field of early childhood interventions in primary and community health and translational research. All authors are female with the first and third authors are coming from CALD background. Thus, the research team on this study has an insider view of health services, which may impact on their perception of women’s views. In order to ensure the authenticity of the women’s view, the research team ensured that the voice of the participants was foremost in the analysis.

**Table 1:** Participants Characteristics

Research participant number	Country of birth	Age	Participants main language	Religion	Number of children	Stay duration in Australia	Completed interview with use of interpreter
P1	Bangladesh	34	Bengali	Muslim	2	14	No
P2	Bangladesh	32	Bengali	Muslim	2	6	No
P3	Bangladesh	34	Bengali	Muslim	2	7	No
P4	Bangladesh	36	Bengali	Muslim	1	15	No
P5	Bangladesh	31	Bengali	Muslim	2	4.5	No
P6	China	33	Mandarin	No religion	2	7	Yes
P7	China	30	Mandarin	Buddhism	1	10	Yes
P8	Morocco	42	Arabic	Muslim	3	7	No
P9	China	30	Mandarin	Buddhism& Christianity	1	14	No
P10	China	34	Mandarin	No religion	2	4	Yes
P11	Taiwan	39	Mandarin	No religion	1	7	No
P12	China	34	Mandarin	No religion	1	10	Yes
P13	China	37	Mandarin	Christianity	3	7	Yes
P14	China	36	Mandarin	No religion	3	1.5	Yes

## Results

Fourteen CALD mothers were interviewed by the first author. All mothers completed the demographic survey; the average age was 34 years (range 30– 42 years) and stay duration in Australia was 8 years (range 1.5–15 years). Mothers’ main languages spoken were Bengali, Mandarin and Arabic (Table 1).

Four themes were identified: managing culture; managing the service system; managing the relationship; strengths and weaknesses of CFHN services.

### Managing culture

This main theme focuses on mothers’ experiences of managing their culture in receiving CFHN services and SNHV programs. Many mothers mentioned that cultural differences had no negative impacts on their mother-nurse relationship, where the differences were not in conflict with practices CFHN promoted, such as breastfeeding.

No, no I never had any problem, they [CFHNs] sometimes they say no, no, you can’t do this, but they [are] strange a little bit, because when I told them I do breastfeeding till 23 months. In Islam it says to [breastfeed] until 2 years, right?! So, I said the nurse I breastfeed him to 23 months, she was little bit surprised but didn’t tell me anything… yes, but they are stick to their rules, but they help you understanding… (P3)

However, some mothers felt that CFHNs only accepted mothers’ cultural practices if there was no conflict with parenting practices in Australia. In such circumstances mothers would follow their cultural traditions about parenting practices and not follow CFHNs advice. This would require these mothers to manage their engagement with the nurse to conceal and thus maintain their cultural practices. For example some mothers would manage the information they shared with the CFHN, such as what food was being introduced to the baby, or manage the timing of engagement with CFHN such as this mother who delayed cutting her baby’s hair until after the nurse had visited.

I’m not sure if you’re stating the cultural things like the hair cutting things is not allowed in Australia, doctor said after three months you can [do] haircut, but in religious matter, I’m not sure you [are] Muslim or not but I’m Muslim in my religious matter 7 days, 21 days or 14 days in between, I should cut the hair but nurses, I’m not sure [if] they’re going to allow it or not. I… after visiting nurse, I got my baby, baby’s hair, so I’m not sure they’re gonna react me or not…obviously, they’ll react because it’s [the baby] three months, they said wait because the skin is very sensitive at the moment so … but I am doing [it] very carefully. (P1)

### Managing the service system

This major theme focuses on CALD mothers’ experiences regarding managing the service system through managing communication; managing interpreter use; using General Practitioners (GPs); and using other resources.

For most mothers, face-to-face engagement with services was preferred as it supported communication and their access to services. Some mothers described that in the face-to-face sessions with CFHNs they use body language to express themselves, which was not possible when offered telephone or group-based services. Mothers reported that they preferred individual face-to-face services as a lack of confidence about parenting skills meant they did not feel comfortable in clinic or group activities. Further joining group activity sessions and calling clinics to book services were problematic due to language barriers.

[I] just [prefer] face to face [sessions with nurses]. Because [in] face to face sometimes, if I want to explain something I [can] use body language…(P8)

Because my language is not good. So, it’s very troublesome to call. That’s why I didn’t call [clinics]. (P10)

Most of the mothers described that SNHV was helpful for in-need and first-time mothers and they also preferred to receive these services at their homes. One mother mentioned that SNHV programs should be compulsory for all mothers and another mother expressed the view that CFHN services should be compulsory for all families as it helps to persuade fathers, who in some cases were unsupportive of the mother seeing the CFHN, to support their use of services for check-up sessions, overcoming barriers to service access.

I think in Australia the nurse must go to home visit for all the babies. I think for all the babies. I think it’s better for the nurse to go home visit. Because if they come, the mother [doesn’t] need to take the baby out. Because mother just gave birth to baby. Mother is still very weak. It’s better not to go out. (P10)

All those compulsory things I think it’s good. It’s good otherwise he’s [husband] never taking it seriously. He [husband] says, they’re [CFHNs] just saying they’re just saying … just leave it [what they say]. Always like that, yeah, it’s good … all compulsory things. (P9)

Most of mothers described that they felt comfortable in using the interpreters provided by the CFHN service and that having interpreters was helpful and supported them to build a good relationship with CFHNs. Six mothers from Muslim backgrounds mentioned they preferred to have female interpreters, however for rest of the mothers there were not any preferences in use of male or female interpreters.

Refuse them [male interpreters] because I feel I am ok, so if I share any confidential issue I don’t want to share [it] because [of] my religious restriction, I don’t want to share it with male interpreter. (P5)

When mothers could not manage to get the face-to-face CFHN service or if they felt no language or culturally appropriate CFHN service was available, many mothers described getting help from GPs instead. Additionally, they reported that they trusted GPs for medical health issues more than CFHNs and took their children for vaccination to GPs. However, this created issues when different advice was received from GPs and CFHNs in regard to parenting practices and child development. Two mothers advised other new mothers to find regular good GPs for their children’s check-ups.

For bigger issues I go to … if they [kids] are sick, I must talk to them [CFHNs] then I go to the GP, otherwise for their regular check-up and if some minor issues like insufficient breast milk and not eating well, or drinking up a bottle of milk not drinking enough water then I go to GP. Yeah, for any issues or vaccines I go to see the GP for my son. (P5)

I think they [mothers] need to like search a lot of information and they can ask about GP, they need to find a regular GP because I didn’t have a regular GP. Only I have regular GP when I had [my daughter]. I found a really good one with a child health. Like, yes… so I think it’s a little bit hard if they don’t have family or friends in Australia. So, they need to do a lot of work to how to raise the baby. (P11)

In the absence of CFHN services, many mothers mentioned they received support from family members such as husband, parents, friends or community, and some mothers highlighted that they tried to get help from an experienced family support (e.g., their parents overseas). Also some mothers mentioned they sometimes tried to search online in their native language to get information about parenting skills due to lack of support from their family, low health literacy about child care and development, and lack of awareness of available services. Some mothers advised new mothers to keep calm and be mindful of self-care and to try and find available services and receive helpful services from other organisations.

I think I really don’t know about the system in Australia. Like, I didn’t know I need to register the childcare a year ago. Because they say… they told me [for] some childcare, you need to wait for maybe few months up to one year to get your child getting to the childcare. And about blue book, I think I only got the blue book when the baby [was] born. If I can get some information before baby’s born, I can prepare some of the … I can have more knowledge. I don’t have to worry too much [to] bring up my baby. (P11)

Be patient, don’t panic, like your mother, you did hard work that’s why she or he [kids] could call you mother. Your mother also sacrificed a lot for you, so you have to be patient, don’t panic. (P5)

### Managing the relationship

This major theme focuses on issues around the nurse-mother relationship. Although many of CALD mothers described that they received support and advice from CFHNs and have a good relationship with them, some issues around the nurse-mother relationship were identified. Some mothers mentioned they had difficulty in understanding bilingual nurses’ accents, making it difficult to engage with the CFHN. Some encountered CFHNs’ strict attitudes in cases where the mother did not follow up their advice. Some CALD mothers mentioned that although CFHNs supported them to increase their confidence regarding parental skills, issues around bilingual nurses’ accents and some CFHNs attitudes forced them to change clinics.

Another nurse asked me to go to the GP, I didn’t go [and] she said she’s going to report on me, yeah, but she was a bit strict. But I couldn’t go to GP by myself at that time, it was too early, like I’m not familiar with how to go and going by myself to GP and my husband always … he’s playing games, so I said to him to go, [he said] let’s leave it, something like this, so then she [nurse] said she is going to report on me. (P9)

My son was not sleeping whole day so I didn’t know what happened to him and he was vomiting a lot and I was very stressed and when she [nurse] told me this is bad, this is bad, you shouldn’t do this you shouldn’t do that, so that time I didn’t understand but now I can realise that, she could talk a little bit nicer, you know, because I was only frustrated. (P3)

Other mothers valued the CFHN service and their relationship with the nurse.

It has given me a great help. And in my mind, I am not anxious anymore in terms of parenting. (P14)

A good thing is good relationship with mothers and nurses could make a good relationship otherwise mothers will not express their problem. (P1)

### Strengths and weaknesses of CFHN services

This major theme focuses mothers’ perception of CFHN service weakness in child development and strength in maternal health and parenting.

Some CALD mothers had concerns about their child’s development and existing medical issues that were not resolving, were not being picked up by the nurse and the GP, or not being followed up well enough. In addition, some mothers described their experiences of being referred to other services by CFHNs, sometimes noting this to be problematic, but for others a positive experience. Some mothers recommended to new mothers to be vigilant and check their babies’ heath and follow up with services with persistence.

My first son just [has] speech issue, my second son [has] autism. Not GP or nurse, in clinic [could find the issue], when I had appointment for first son, the Dr [Speech Pathologist] found and said I want to see this boy, and yeah… (P8)

Especially I have this problem with my son, … [they put] my son and me in waiting list for so long, sometimes I don’t know how to help them [my children] yeah, … sometimes just I go to my phone to YouTube. I look what I [can] do and find [in my own language]. Something [I am] just one help to myself. (P8)

Many of the CALD mothers described positive impacts on their child-mother relationship from receiving CFHN service and SNHV program. In addition, some mothers mentioned that the CFHN service helped mothers to learn new things and be better mothers for their older or younger children.

I think it’s good, it’s great [help], I don’t know how to express myself, and I think they [CFHNs] are … they taught me how to feed my first child and how to care about my first child and taught me a lot. (P6)

In addition, some mothers described that they experienced mental health issues, domestic violence and suicidal feeling, and three mothers mentioned they received good mental health support and helpful maternal advice from CFHNs and social workers.

I think in Australia, always care about mum’s mental health issue. They [CFHNs] always ask me how you [are] doing and then I think it’s very different from my country. I think it’s very good and positive to do that … yeah. (P11)

I …so long story. We were on to the lot of difficulties, for myself and children’ father and at one stage of our life [we] didn’t afford accommodation, we can’t afford a rent and they [nurses] helped me a lot and then [in] another stage of life when I had my second child I had a lot of arguments with the children’ father and I was very depressed at that time they referred to social worker and other services. I think they made a lot of difference in my life. And even I thought of death at that time. They really helped me a lot. (P6)

Overall, mental health and parenting support were the areas where mothers felt the CFHNs were most consistently helpful, however, for a number of mothers, there were concerns about the CFHNs management of their child development concerns.

## Discussion

This study describes the lived experiences of CALD mothers with young children who were delivered CFHN services and SNHV programs through a socioecological lens. The principal findings were categorised into four themes: managing culture; managing the service system; managing the relationship; strengths and weaknesses of CFHN services. These themes, and the superordinate concept of ‘having to manage’ related to two levels of the socioecological model: institutional, and interpersonal issues.

The study findings indicate that there are institutional-individual relationship level service system issues that constrained effective engagement of CALD mothers with CFHN services. CALD mothers, often in the absence of clear knowledge about health system, barriers to communication and access to CFHN services in the way they wanted, particularly face-to-face at home, were trying to manage the service system that was not always supportive of their language and cultural values. They managed by visiting female GPs from the same cultural background when female interpreters were not available, searching health resources online in their own language, and seeking support from family. These findings are consistent with previous studies indicating how CALD mothers managed to communicate as best they could by visiting GPs from the same cultural and language background to avoid misunderstanding of any health-related information that could compromise their children’s health [6, 20]. The findings also showed that low health literacy was intensified by language, cultural and trust relationship issues and contributed to inequities through impeding CALD mothers’ access to and engagement with the services. Previous research has similarly underlined the significance of low health literacy and highlighted providing information in native languages increases health literacy and is desired by CALD families [21]. Furthermore, results of this study identified that although cultural differences have no negative impact on many of CALD mothers’ relationship with CFHNs, some mothers stated that they follow their own cultural parenting practices if there is a conflict with parenting practices offered by CFHNs. This supports previous meta-ethnographic research that culturally appropriate services are more likely to be used and suggested by migrant women, and a lack of sensitivity to cultural practices reinforced a disconnect between migrant women and healthcare professionals [3].

It was also apparent that the interpersonal level mother-nurse trust relationship is a core factor in access to and engagement with CFHN services and SNHV programs [9]. The CALD women in this study identified some issues around nurse-mother relationships they experienced, particularly those with LEP, who found nurses accents difficult to understand. CALD mothers were interested in their child’s development and managed their own way to build a good relationship and better access and engage with the CFHN services (e.g., changing clinics) such as avoiding nurses ‘strict attitudes’ about parenting practices, care and responses to advice. However, these challenges towards mothers were impeding factors that broadly could lead CALD mothers to avoid or delay accessing and engaging with the services. Although many mothers described improvements in their child-mother relationship and receiving good mental health advice by CFHNs, some mothers had concerns around child development and perceived those as being neglected by the service system. This finding is well supported by previous Australian research that CALD families’ concerns about their children’s developmental issues were not being identified and that there are problems accessing early intervention in a timely manner [5]. In agreement with a previous study, nurses’ respectful and companionate manner can increase the trust relationship and support mothers’ efficient engagement with the services [22].

Findings also revealed that although CALD mothers face difficulties in accessing and engaging with CFHN services, the resiliency of CALD mothers was revealed in their positive strategies in managing and coping in the face of difficult circumstances, for example, by managing the sharing or not sharing of cultural practices, managing the service system through seeking alternative sources of support and information, or managing the nurse-mother relationship by changing to a different CFHN clinic. However, the additional burden placed on CALD women to manage their culture, the system, and their relationship with the CFHNs when engaging in services may be contributing to the inequities of access and engagement noted here and in other studies [3–6]. This may be understood within the ‘dominant cultural expectation of agency in the Western world’ [23], where it is expected that individuals are responsible not only for their own health and well-being, but also for negotiating the comfort of the services with whom they engage. There is evidence in this study that the mothers feel that the responsibility for service engagement is placed on individuals while the service systems, which have significant impact on individuals’ health, are not, in some cases, able to fulfil their responsibilities to maintain and provide equitable and culturally inclusive environments [23].

This research shows that it is important to put strategies in place that allow these vulnerable families to engage effectively with CFHN services. Consequently, to improve health outcomes for CALD families with LEP, reduction in inequities in the burden of responsibility as a fundamental element in healthcare systems, particularly in the context of relationship-based services, is required. For example, to achieve greater equity, promoting service responsibility to create a more equal power balance in the CALD mother-nurse relationship, such as exists in SNHV models; better understand families’ cultural differences; identify and assume the need for female interpreters; and provide translated resources, may improve CALD mothers’ access to and engagement with the services.

### Strengths and limitations

The strength of this study is in highlighting lived experiences of CALD mothers with young children with LEP who received CFHN services and SNHV programs. This study was conducted in two LHDs in areas with substantial CALD populations in Sydney, New South Wales (NSW). However, only a small number of CALD mothers who received CFHN services and SNHV programs with three distinct LEP populations were part of this research, limiting transferability of these findings to other services or cultural groups. Furthermore, conducting interviews with some mothers with assistance of interpreters face-to-face or via teleconferences, and some mothers without using interpreters upon their request should be noted as limitation of this study. As it could be contributed to apprehension in the interview situation and loss of non-verbal interaction. Also, participants’ responses were recorded and transcribed in English only due to cost and time constraints. Nevertheless, the study results provide some significant insight into existing access and engagement challenges and difficulties that would be relevant for other similar CALD communities in high income countries.

## Conclusion

The study describes the lived experiences of CALD mothers with LEP who live in Sydney, NSW, and how they perceive and make sense of their experiences of receiving CFHN services and SNHV programs. The findings indicate a great need for high-quality models of care to be designed and implemented that are responsive to the needs of CALD mothers, to pave a way for these populations to better access and engage with healthcare services. These mothers with young children, particularly with the added vulnerability of LEP, were having to take the responsibility to manage their culture, the service system and their relationships with healthcare professionals in order to receive support in the way they desired.

## Electronic supplementary material

Below is the link to the electronic supplementary material.

Supplementary Material 1

## Data Availability

The datasets generated and/or analysed during the current study are not publicly available due to the sensitive nature of the questions asked in this study and confidentiality but are available from the corresponding author on reasonable request.

## Footnotes

1 In the Australian context, culturally and linguistically diverse is a widely used term that refers to those from non-English speaking countries and with specific cultural practices and values.

## List of abbreviations

CALD: Culturally and linguistically diverse
CFHN: Child and family health nursing
CFHNs: Child and family health nurses
IPA: Interpretative Phenomenology Analysis
GPs: General practitioners
LEP: Limited English proficiency
LHD: Local Health District
NSW: New South Wales
SESLHD: South Eastern Sydney Local Health District
SLHD: Sydney Local Health District
SNHV: Sustained nurse home visiting.
