# Supplementary material for: Having to manage: culturally and linguistically diverse mothers’ lived experiences with sustained nurse home visiting programs
Source: BMC Health Serv Res. 2023 Apr 11;23:354. doi: 10.1186/s12913-023-09315-9 (PMC10091528; doi:10.1186/s12913-023-09315-9)
Supplement: Supplementary file 1 — Supplementary Material 1 [file 12913_2023_9315_MOESM1_ESM.docx]

**Appendix 1: Family Interview Questions**

1. Thinking back to when you first came home from hospital with <CHILD> and the first 8 weeks what were some of the things you found useful in helping your parenting?
2. How about after you had some time to adjust and get to know your child? How was the first six months? The first year? (PROMPT: what did you find difficult or useful? –Parenting skills)
3. What did you do when you wanted some advice or help with your child?

(PROMPT: Where did you go for help? Who did you speak to?)

1. What other sources of support did you have? (Have you received any support from family and/or community?)
2. From what you have learnt what advice would you give new parents?
3. What was it like when the nurse visited?
4. In what ways did the visits help you?
5. In what ways were they unhelpful? (PROMPT: Older/younger children, first time parenting, difference in what you imagine you would do – what do you think might have happened if the nurse had not visited?)
6. What sort of differences did the visits make?
7. Did it make a difference in how you helped or parented your child?
8. Did it make a difference in how you think of yourself as a parent?
9. Did it change your relationship with your child? (PROMPT: did you feel you could better understand your child or respond to their needs?)

1. How did you get on with the nurse?
2. How did you feel when the nurse visited?
3. Did this change over the year?
4. Did you feel the nurse was sensitive to your culture?
5. What sorts of things did the nurse do that made you feel this way?
6. Were there times that you felt cultural differences or a lack of understanding about your culture caused problems or impacted on your relationship with the nurse?
7. Could you please describe these times for me? [PROMPT – what happened? Why did you feel this way?]
8. How did you respond or react during these times?
9. Is there anything the nurse could have done differently? Is there anything you could have done differently?
10. Was it helpful to have the interpreter at the home visits?
11. Did you feel comfortable using the interpreter?
12. Did this change over the year?
13. Did you think having the interpreter impacted on you building a relationship with the nurse?

1. What advice could you give new mothers about having a nurse home visit?

(PROMPT: what would you have liked to have heard before you had a home visit?)

1. What do you think should happen with home visits?
2. For example, should they be for everyone, just people with difficulties, more often, just one off, longer, shorter etc

1. What would you change about the home visiting program?
2. How do you think this could help?

1. What would you have done without the intervention? (PROMPT: Where do you think you would have gone for help?)

(If necessary, ask: And could you tell me who currently lives with <CHILD> and yourself?)

**For mothers with other children (multiparous) mothers only (Questions 15, 16) ONLY**

1. How did the visits change how you parented your older child/children?
2. How did the visits change how you parented your younger child/children?
